# Supplementary material for: Case-control investigation of invasive Salmonella disease in Malawi reveals no evidence of environmental or animal transmission of invasive strains, and supports human to human transmission
Source: PLoS Negl Trop Dis. 2022 Dec 12;16(12):e0010982. doi: 10.1371/journal.pntd.0010982 (PMC9779717; doi:10.1371/journal.pntd.0010982)
Supplement: S1 Fig — (PPTX) [file pntd.0010982.s002.pptx]

## Slide 1
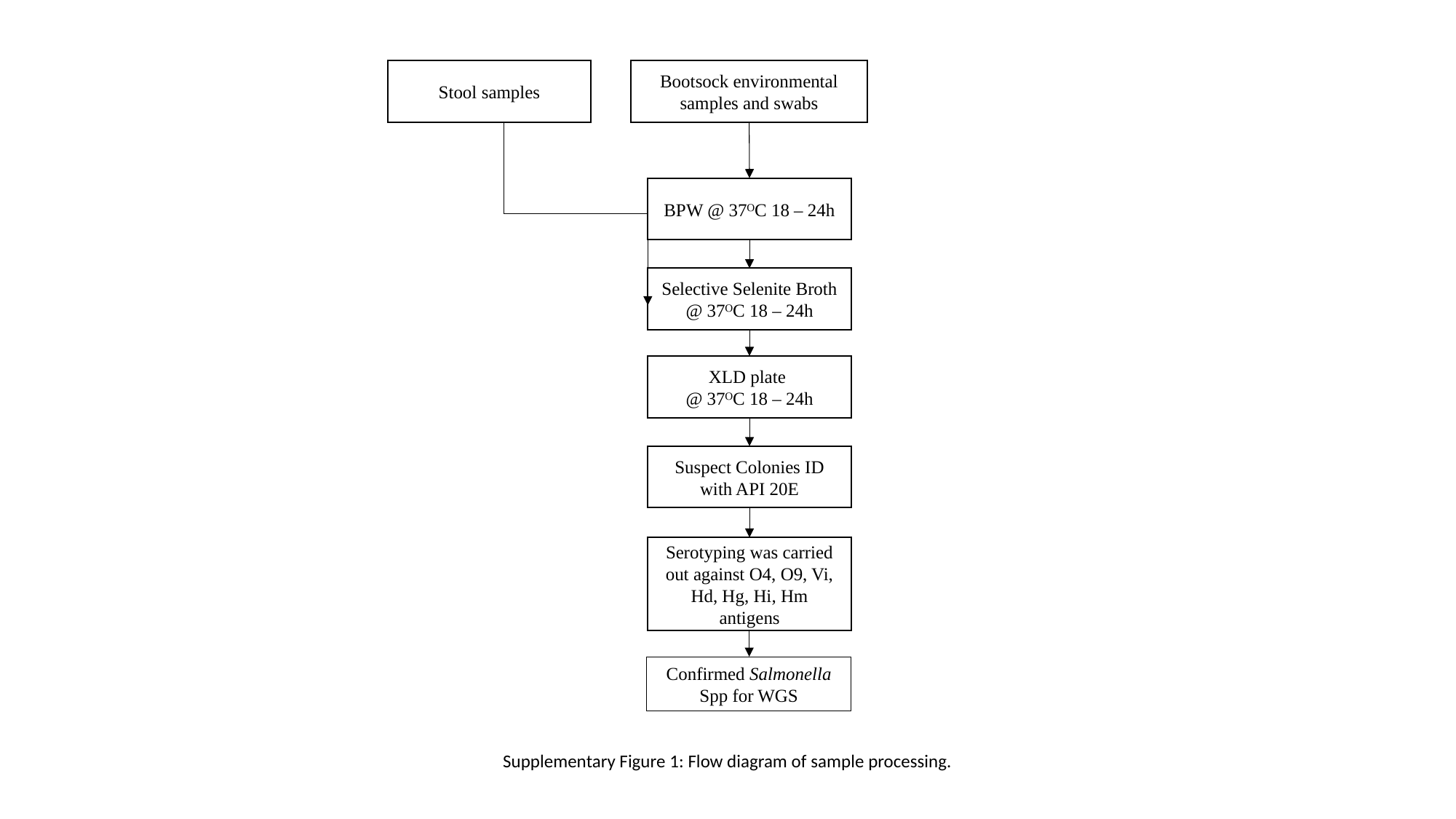

Stool samples
Bootsock environmental samples and swabs
BPW @ 37ᴼC 18 – 24h
Selective Selenite Broth @ 37ᴼC 18 – 24h
XLD plate
@ 37ᴼC 18 – 24h
Suspect Colonies ID with API 20E
Serotyping was carried out against O4, O9, Vi, Hd, Hg, Hi, Hm antigens
Confirmed Salmonella Spp for WGS
Supplementary Figure 1: Flow diagram of sample processing.
