# Supplementary material for: Case-control investigation of invasive Salmonella disease in Malawi reveals no evidence of environmental or animal transmission of invasive strains, and supports human to human transmission
Source: PLoS Negl Trop Dis. 2022 Dec 12;16(12):e0010982. doi: 10.1371/journal.pntd.0010982 (PMC9779717; doi:10.1371/journal.pntd.0010982)
Supplement: Attachment — Submitted filename: PNTD-D-22-00359_Rev.pdf [file pntd.0010982.s003.pdf]

Case-control investigation of invasive Salmonella disease in Africa reveals no evidence of environmental or animal reservoirs of invasive strains

Koolman et al present data on *Salmonella* isolates collected from people, household members, animals, and the household environmental of invasive *Salmonellosis* cases and their matched controls households in Blantyre, Malawi. They report on both invasive non-typhoidal *Salmonella* and typhoid cases and matched controls. They also report sequence type, serovars, and serogroup. Finally, they present results of whole genome sequencing of the *Salmonella* isolates collected. The study design, robust and novel sampling, and the clinical and public health importance of this issue make this a valuable contribution to the literature. However, some key study concepts need to be clarified in this manuscript in order to maximize the impact of this study. Addressing these and other concerns listed below would greatly improve this important work. Additional minor concerns also listed below.

MAJOR EDITS/CONCERNS

My primary concern with the manuscript is how the study outcome has been described and written about. It appears the authors enrolled iNTS and typhoid cases, that is, patients with iNTS and typhoid disease (see lines 31, 107). However, iNTS is also referred to as a cause of infection rather than the result of infection (see lines 120, 374, 441-442). For example, I believe line 374 should say humans are the primary reservoir for iNTS <u>*causing*</u> *Salmonella*, not reservoir for iNTS. Please use terminology consistently throughout the manuscript, being explicit about what is the pathogen/etiology versus the disease/symptoms. The Introduction should be edited to clearly explain the relationship between these terms, as well as to introduce the concepts of sequence type, serovar, and serogroups of *S.*, and to describe the symptoms and clinical presentation of iNTS and typhoid versus non-invasive *S.* infection. Also, please be sure to update the study aims listed in lines 128-134 appropriately. See also lines 78-82, 260-261.

MINOR EDITS/CONCERNS

Please define abbreviations at first use.

Line 27: isolates from symptomatic and healthy people

Line 92-94: Add additional detail on incubation period, how long S. typhi survives in environment, etc.

Line 109: environmental factors could also lead to seasonal patterns that have nothing to do with animal reservoirs, e.g. more person-to-person contact during cold weather and therefore increased person-to-person transmission.

Line 128: Authors list the study goal as <u>identifying the reservoir</u> of Salmonella strains responsible for invasive disease. The authors do not list this, but this study also answers the more

fundamental question of what strains of Salmonella are people infected with when they have iNTS and typhoid symptoms/disease?

Supplemental figure would benefit from footnotes or other method to provide additional information about abbreviations.
