# Supplementary material for: Case-control investigation of invasive Salmonella disease in Malawi reveals no evidence of environmental or animal transmission of invasive strains, and supports human to human transmission
Source: PLoS Negl Trop Dis. 2022 Dec 12;16(12):e0010982. doi: 10.1371/journal.pntd.0010982 (PMC9779717; doi:10.1371/journal.pntd.0010982)
Supplement: Attachment — Submitted filename: 2022.08.26 Response to reviewers MG PA.docx [file pntd.0010982.s004.docx]

Dear Dr Ashton,
 
Thank you very much for submitting your manuscript "Case-control investigation of invasive Salmonella disease in Africa reveals no evidence of environmental or animal reservoirs of invasive strains" for consideration at PLOS Neglected Tropical Diseases. As with all papers reviewed by the journal, your manuscript was reviewed by members of the editorial board and by several independent reviewers. In light of the reviews (below this email), we would like to invite the resubmission of a significantly-revised version that takes into account the reviewers' comments.

We cannot make any decision about publication until we have seen the revised manuscript and your response to the reviewers' comments. Your revised manuscript is also likely to be sent to reviewers for further evaluation.

 When you are ready to resubmit, please upload the following:

[1] A letter containing a detailed list of your responses to the review comments and a description of the changes you have made in the manuscript. Please note while forming your response, if your article is accepted, you may have the opportunity to make the peer review history publicly available. The record will include editor decision letters (with reviews) and your responses to reviewer comments. If eligible, we will contact you to opt in or out.

[2] Two versions of the revised manuscript: one with either highlights or tracked changes denoting where the text has been changed; the other a clean version (uploaded as the manuscript file).

Important additional instructions are given below your reviewer comments.

Please prepare and submit your revised manuscript within 60 days. If you anticipate any delay, please let us know the expected resubmission date by replying to this email. Please note that revised manuscripts received after the 60-day due date may require evaluation and peer review similar to newly submitted manuscripts.
 
Thank you again for your submission. We hope that our editorial process has been constructive so far, and we welcome your feedback at any time. Please don't hesitate to contact us if you have any questions or comments.
 
Sincerely,

Andrew S. Azman
Deputy Editor
PLOS Neglected Tropical Diseases

# Reviewer #1:

## Summary:

The study provides additional insights to an important knowledge gap, i.e. transmission of iNTS in SSA. It strenghtens the hypothesis that non-typhoidal Salmonella in sub-Saharan Africa have adapted to the human host and human-to-human transmission.

**Authors’ response:** We thank reviewer 1 for noting the importance of this work, which advances an important area in the field with fresh data and insights.

Reviewer 1: Major comments (see section-specific comments) on the study and manuscript are :
- The manuscript is relatively long, contains redundancies and might benefit from restructuring. Some sentences should be rephrased to improve clarity. Avoid discussion of the data in the results section. Review by one of the native English speaking co-authors is required.

**Authors’ response:** We note these comments, also reflected in some comments from other reviewers. We have reshaped some components of the manuscript and relocated material from introduction to discussion, to streamline and focus the material. The manuscript has been both written and reviewed by native English speakers.

Reviewer 1: - A flow chart clarifying patient and household enrollment and sampling should be added, including a brief sociodemographic description of cases and households.

**Authors’ response:** a new table demonstrating demographics and sampling for each category has been included (new Table 1).

- A map should be added to display the geographical distribution of households.

**Authors’ response:** we thank the reviewer for this helpful suggestion, and have mapped the data by area (new Figure 1), creating a double panel mapping of households recruited (indicating numbers of Salmonella-positive and Salmonella-negative households), and of the geographical distribution of Sequence Types in those houses, colour coded my sample-type for clarity. This demonstrates that our investigation evenly covered a broad area of town, and also supports our comments on geographical distribution of sequence types. We agree this is a very helpful addition, and include this as a new figure.

Reviewer 1: - Line 48: "healthy humans were the source of iNTS infections in the household". The study results support indeed the hypothesis of human to human transmission, albeit only based on 2 pairs. This association however does not allow statements on the source of infection/direction of transmission. I also suggest to integrate the hypothesis on a common source of infection in the abstract conclusion.

**Authors’ response:** Reviewer 1 is misquoting the actual sentence in the abstract conclusion at line 48. We do not claim that “humans were the source of iNTS infections in the household”.

In the sentence in question, we state that **“The finding of NTS strains from index cases that matched household members, coupled with lack of related animal or environmental isolates,** ***supports a hypothesis* that healthy humans were the source of iNTS infections in the household.”** (italics for emphasis here). It is clear elsewhere in the manuscript that we do not consider this hypothesis to be proven in absolute terms by our data, since it is impossible to prove without a longitudinal tracking component to conclusively demonstrate transmission pathways.

In this study we have made a more comprehensive search in the environment and among household animals than any previous study for a “common source”, and not found one despite very representative sampling and a good yield of a wide range of Salmonellae in all sample categories. We also make this point in the very same sentence in the abstract, making the nature of the evidence very clear, which the reviewer has omitted to take account of.

We will, however, modify the particular sentence in the abstract, to read “…… supports a hypothesis of human to human transmission”, since “source” perhaps has a more limited technical meaning. The abstract now reads:

**“****The finding of NTS strains from index cases that matched household members, coupled with lack of related animal or environmental isolates, supports a hypothesis of human to human transmission of iNTS infections in the household.”**

We have also added this wording to the title

Reviewer 1: - Line 51 - 53: In addition to the absence of Salmonella Typhi isolated from the environment, the zero positivity from human faeces is remarkable and might indicate limited sensitivity for isolation of iNTS from faecal samples too.

**Authors’ response**:

We do not find the absence of S. Typhi from stools of healthy family members at all remarkable, it is exactly what we would have anticipated from the existing literature. The low rate of detection of S. Typhi from humans reflects well-recognised differences in epidemiology and microbiology, rather than any deficiency in our microbiological methods:

The current understanding and experience of typhoid transmission is that stool shedding would be either in the stool of very rare but chronic carriers, or else much more transient shedding from convalescent cases. The reviewer may note that we did NOT take convalescent stool from any of the index cases themselves (either typhoid or iNTS cases) from whom we might have anticipated to isolate S Typhi, as this was not integral to the aims of the study or part of the protocol. In our earlier STRATAA community surveillance typhoid study (Meiring et al, https://www.thelancet.com/journals/langlo/article/PIIS2214-109X(21)00370-3/fulltext), we found no stool-positive cases in the community suggesting a vyer very low rate of chronic carriage, and in all similar previous published papers from our setting that are cited in this manuscript, there are similarly low detection rates of S. Typhi in the community compared to NTS.

In contrast, asymptomatic carriage of a range of NTS serovars in healthy humans is known to be common in low-income settings. The literature reported in this manuscript universally suggests a rate of 3-6% and this accords entirely with our findings in this study, a point we make in the manuscript.

To illustrate the contrast between healthy carriage rates of typhoid and iNTS: in a current community-based study of children in Malawi, using well-validated and optimised selective stool microbiology methods for Salmonella stool culture similar to those in this study (selenite broth and XLD plates), we have sampled 2424 healthy children, and detected 125 varying NTS isolates (5% isolation rate), but just 2 typhoid isolates (0.08% isolation rate), despite this survey being conducted in a setting where both iNTS disease and typhoid are known to be endemic.

In relation to the lack of detection of S. Typhi from the environment compared to NTS: The technical challenges that we refer to with respect to growth of *S.* Typhi from environmental samples are well recognised and relate to the possibility of the organism entering a viable but non-culturable state. This is biologically distinct to S. Typhi rather than other NTS serovars, and is an area of pathogen biology discussed in detail in the reference provided, but the detail is beyond the scope the manuscript.

It is striking that we also have a similar recovery rate of NTS from a range of environmental samples in addition to human stool, suggesting that our well-validated microbiology methods are sensitive and appropriate for NTS across a range of sample-types.

In short, we do not believe that our culture methods limited our detection of NTS Salmonella serovars. The relatively low detection of S Typhi compared to NTS from either humans is due to fundamentally different disease pathogenesis and epidemiology, in a highly human-adapted microorganism The different isolation rate from the environment may be due to underlying differences in basic pathogen biology which have no bearing on NTS.

We have accordingly affirmed the robustness of the culture methods in the abstract, and made clearer specific mention of the lack of healthy carriage of typhoid, as well as absence from the environment, as below (italics are new additions):

**“The breadth of NTS strains found in animals and the household environment across all sites demonstrated the robustness of NTS sampling *and culture* methodology, and suggests a diverse ecology of *Salmonella* in this setting. *Healthy typhoid (S. Typhi) carrier state was not detected*, and the lack of *S.* Typhi isolates from the household environment suggests that further methodological development may be needed to culture *S*. Typhi from the environment.”**

## Introduction:

Reviewer 1 comment: - The introduction is long and its readability and storyline can be improved. Please restructure the paragraphs, so that each paragraph focusses on one topic.
- Information on NTS in HICs vs typhoid fever vs iNTS is a bit scattered across the paragraphs. To help the reader, it might help to focus on main concepts, e.g. "NTS in HICs = zoonotic transmission, intestinal infection" vs. "typhoid fever = human-to-human transmission, invasive disease" vs "iNTS= transmission not fully elucidated yet but human-to-human transmission hypothesized, invasive disease".

**Authors’ response**:

Thank you for the comment, we accept this and have amended the manuscript, as above, to explain the differences between the 3 clinical presentations as suggested, and improve the focus and flow. We have outlined previous literature in the introduction in order to frame the hypothesis and study design, but moved detailed comment on previous literature to the discussion, and reshaped to avoid repetition.

Reviewer 1 comment: - Available evidence on iNTS transmission is discussed in the introduction and discussion section. Suggestion to keep in-depth discussion of transmission for the discussion section and only briefly refer to possible transmission routes in introduction. When discussing available evidence, please group evidence as much as possible by hypothesis, e.g. all data in favor of human-to-human transmission together. It is a bit hard to follow now due to jumping back and forth between isolates from animals, healthy children and the environment.

**Authors’ response**:

Thank you for the comment, as above, we have amended the manuscript, as above, to improve the focus and flow. We have outlined previous literature in order to frame the hypothesis and study design, but moved detailed comment on previous literature to the discussion, and reshaped to avoid repetition.

## Methods:

Reviewer 1 comment: - Line 145-146: Are predefined criteria for suspicion of sepsis used?

**Authors’ response**: There are several international clinical definitions of sepsis, which may vary between adults and children, and recognition of sepsis at QECH is at the admitting physician’s judgement, but includes (in addition to the over-arching criterion of fever) evidence of circulatory failure including hypotension, signs of peripheral shut-down, reduced consciousness and end-organ failure. Any of these may trigger a blood culture being taken.

Reviewer 1 comment: - Line 152: "with recurring iNTS disease were excluded". Do you mean deduplication or first presentation also excluded?

**Authors’ response**: Individuals with HIV are known to be prone to recurrent episodes of iNTS – indeed recurrent iNTS was one of the original clinical criteria defining end-stage HIV disease. Our previous research has demonstrated that these recurrent episodes (happening within 30 days) are usually caused by recrudescence of an identical strain (Gordon et al, AIDS 2002; 16: 1633-1641. PMID:12172085; and Okoro et al, Clinical Infectious Diseases 2012; 54: 955-63. PMID:22318874), and that this identical strain likely re-emerges from a site of intracellular sequestration (Gordon et al, Clinical Infectious Diseases 2010; 50: 953-962. PMID:20180702).

This means that, although recurrent iNTS events are extremely rare compared to primary iNTS events, their source is likely to be intrinsic rather than extrinsic. For this reason people with recurrent iNTS episodes were excluded from the search for an extrinsic source. A first ever presentation would not be excluded, only recurrent/recrudescent presentations within 30 days.

In practice, however, recurrence / recrudescence is nowadays much rarer due to Malawi’s high coverage with effective ARTs among people living with HIV, and we did not need to exclude any recurrent/recrudescent events from recruitment, as none presented during the course of the study.

We have changed the term “recurring” to “recurrent” for clarity, and added the clinical definition of recurrence (a known blood culture positive episode in the previous 30 days). We have added the information that no cases were excluded for this reason in the results section

Reviewer 1 comment: - Line 155: Sociodemographic data were collected. Please provide some basic sociodemographic data here (including household size and age of household members).

**Authors’ response:** A table of demographics including household size and age groups for each category has been included (as noted above, see new Table 1). This demonstrates that sampling and isolate retrieval rates were well balanced between case and control households, and that the rate of sampling between adults and children was similar between cases and control and reflected the demographics of the general community.

Reviewer 1 comment: - Line 157: GPS coordinates were collected. To allow insights in the geographical distribution of households and geographical interrelatedness of isolates, I strongly suggest to provide a map displaying all sampled households and isolation of iNTS.

**Authors’ response:**

This was a very helpful suggestion for which we thank the reviewer. We have created a panel of a pair of maps (see new Figure 1), showing the firstly the distribution of sampling and salmonella retrieval across wards of the city, and secondly the distribution of each Sequence Type by ward of the city, colour-coded by source of isolation to help interpretation and reduce the need for cross-reference to tables. This demonstrates that our sampling and rate of isolation were both very evenly distributed across the city, and gives visually-accessible support to our comments on relatively localised geographical distribution of sequence types. This is now included as a figure.

Reviewer 1 comment:- Line 158-159: "Exclusion criteria for control households were current treatment for invasive Salmonella disease for any family member" Were there any household members from index cases who were receiving antibiotics?
Also exclusion if antibiotic treatment ongoing for an illness not caused by Salmonella?

**Authors’ response:** The methods section clearly states that household members were only excluded if they were taking antibiotics for a recent episode of invasive Salmonella disease. The reasoning was to prevent falsely-low detection rates in stool from family members, because of treatment using an antibiotic to which their Salmonella was known to be susceptible. This exclusion only pertained at an individual level. Individuals were not excluded for taking antibiotics for any other reason, since ST313 are multidrug resistant, and there would be no way of knowing the susceptibility of Salmonellae for the particular antibiotic in use.

We are not sure why the reviewer did not find this clear, but have slightly rephrased, to make it clear that this pertained at individual level, as below:

“Exclusion criteria for individual household members were current treatment for recent invasive *Salmonella* disease, or declining consent”.

In practice, no household member was found to have had recent episodes of invasive Salmonella disease or to have been prescribed antibiotics, and thus no household member was excluded for this reason. This is now added to the manuscript

Reviewer 1 comment:- Line 169: "Available household members". Please be more specific: at home during the visit? what was the coverage: how many household members out of all household members were sampled? was there a difference in coverage between adults and children?

**Authors’ response:** “Available household members” refers to household members (adults and children) who were willing and able to provide at least one stool sample

in the 24-28 hours during which they were left with stool containers. This did not depend on them being present in person during the actual study visit contact-time. We have clarified in the text, as below:

“Sample containers were provided 24-48 hours before the main sample-collection visit, and we requested that up to three consecutive stools were collected from each individual in the household who was available during that time period.”

We were not able to record individual household members who were “available” but did not provide a stool sample. However, we know from contemporaneous community census data in Blantyre that the average household occupancy is 4.3 people (Meiring et al, 2021 Lancet Global Health PMID: 28674145). Based on this, we would predict 528 “available” people in 60 households, and we actually obtained 491 samples, suggesting that 93% of predicted total possible samples were collected (range 81%-113% of predicted in each category), indicating good overall coverage.

From the same census, we would expect 48% of the community to be aged less than 18, the actual percentage from children was 46.6%, again suggesting representative coverage of sampling between adults and children. This information has been included in the new demographic table and in the manuscript.

- Line 199: Suggestion to move sentence "Serotyping was performed by the slide agglutination technique." to line 203 (after discussion of subculturing).

That improves the flow, thank you.

- Supplementary Figure 1 does not specify the place of serotyping in the laboratory work-up.

Thank you for pointing out this omission, we have corrected it.

- Line 224: All serotype data in the paper were inferred from WGS and phenotypic serotyping only used to decide whether or not the isolate should be shipped to the UK (line 209)?  Please clarify.

Apologies if it was not clear, initial serotyping was done in Malawi, as mentioned in the methods (and now added to the lab work flow chart). The serotype reported in e.g. Supplementary Table 1, and elsewhere in the manuscript, was derived from the genome sequence.

## Results:

Reviewer 1 comment: Please include a flow chart/table to clarify enrollment, yield and characteristics of index cases and households: specify total patients eligible (blood culture sampled), NTS and S. Typhi blood culture positivity, enrolled households, household size, number of samples per household member, age of household members (sampled). If possible, additional data on household members would be interesting to provide more insights on fecal carriage: median interval between sampling and clinical presentation/fever start,  presence of fever/gastro-intestinal symptoms in household members and antibiotic treatment.

**Authors’ response:**

Thank you for requesting these clarifications. Several of the suggested parameters are already included/available. The beginning of the results section already clearly states the number of eligible case households that were approached but declined participation (7 declined out of 67 approached, 3 typhoid and 4 iNTS), and we do not feel that this simple information merits its own flow-diagram.

The number of enrolled households are tabulated, and number of samples per household already are clearly apparent from the current table 1. Other descriptors suggested have now been included in a household demographics table.

Because the sampling protocol was very intense and required significant time in the field, we could not recruit every blood culture positive case. We therefore systematically recruited the first 2 sequential cases, one each of typhoid and NTS, each week. On the few weeks when there was no NTS case, we replaced with a case of typhoid instead. We have clarified this in the Method section.

We have further clarified the protocol requirement for a maximum 14 day interval from index case presentation to household sampling. However, requesting “median interval between sampling and clinical presentation/fever start” is perhaps a misunderstanding of the protocol – no stool samples were taken from symptomatic subjects, but only from their healthy household members.

- Line 256: Not clear what you mean with "similar accros different household categories", because the yield varies from 10-34%

The reviewer is mistakenly referring to *household* positivity according to category.

The text reads “Overall, ***sample*** positivity was similar for human, animal and bootsock environment samples (human stool 16/491, 3.3%; animals 4/110, 3.6%; bootsocks 23/620, 3.8%), and across different household categories (Table 1)”. (our italics for clarity)

This statement clearly refers to *sample* positivity, which is 3.2%, 2.3%, 3.2% and 5.5% across the 4 household categories. We have therefore made no change the manuscript.

Reviewer 1- Line 259 - 261: "Salmonella bacteria ... household samples". Suggestion to integrate these sentences in first part of paragraph describing overall yield.

Thank you for this pertinent suggestion, we have made this change.

Reviewer 1: - Table 1: Please provide data on the proportion of household members from whom NTS were isolated, if possible stratified according to age.

**Authors’ response**:

Isolation rates by household and by sample-type are already included in a table.

The specific split of sampling and of positivity between adult/child samples is now included in the new demographics table, as above. This demonstrates that the proportion of samples from adults and children was equal between case and control groups, and also closely reflected the demographic distribution in the general community.

Reviewer 1: - Table 2: Please specify that serotypes were inferred from WGS in the table header.

Table 2 from our initial submission is now combined with Supplementary Table 1. We have made the clarification you requested on Supplementary Table 1.

Reviewer 1: - Table 2: Suggestion to add to the table caption that the table only contains information on Salmonella "resuscitated from the freezer"

As Table 2 is now combined with Supplementary Table 1, we feel this comment no longer applies. We think that the caption for Supp Table 1 is clear that the results presented are only for sequenced Salmonella.

Reviewer 1: - Table 2: Last column is same as first column.

**Thank you for noticing this error,** Table 2 is now combined with Supplementary Table 1, and no longer contains the duplicate column.

- Line 280 - 285: "There were two instances .... from their stools (Figure 1). Suggestion to rephrase, not clear that first and second sentence are about the same 2 pairs.

**Authors response**: This has been amended as part of the restructuring of these sections

- Figure 1: What does the dark red color refer to?

This has been corrected, it was a display/printing error with the software used to generate the minimum spanning tree.

- Line 305: ST325 is not mentioned in Table 2 or Figure 1

This was an error, this should have been ST316. This has been corrected.

- Line 308 -310: "Some STs isolated ... human or animal isolates.": Already described in Table 1 and previous paragraph, isn't it?

**Authors response**: we discussed these results first by considering molecular sequence-type, then by considering predicted serovar name. This is the because of old-style and new-generation microbiology terminology are both in common use, and different “audiences” prefer each! We agree that this approach has made it somewhat repetitive, and have therefore restructured to discuss ST and serovar alongside each other rather than sequentially. The relationship between ST and predicted serovar is also clearly detailed in a table for reference.

- Line 336: "This indicates a very marked geographical diversity between households". I don't fully understand what you mean and don't see how the data shown allow statements on geographical diversity. See comment above on providing a map with geographic distribution of sampled households and isolates.

**Authors’ response:** Thank you for this very helpful suggestion. As above, we have created a panel-figure with a pair of maps showing the geographical distribution of sampling, positivity and of sequence types across all wards. This shows that sampling and isolation was well-distributed across the whole city, but many serovars are geographically restricted to a single household/ward, and we now refer to it in the text at the appropriate points. This complements the detail from the tables very well.

Reviewer 1:- Line 361: The paragraph on AMR is a bit out of the scope and methodologically relatively weak due to the absence of phenotypic testing. If kept in the manuscript, I strongly suggest to be more specific: which molecular mechanisms were assessed but not present, provide numbers of isolates in which each mechanism was retrieved (instead of "all or some of the isolates") and add for each mechanism the antibiotic (class) to which it confers resistance. Is the presence of fosA7 gene clinically/epidemiologically relevant?

**Authors’ response:** Thank you for this suggestion. We agree that it is out of scope and does not contribute to addressing the hypothesis and aims of the paper. We have removed this paragraph but left the supplementary table, and simply made reference to the table in the whole genome phylogeny section, should some readers have a specific interest in this. The full genomic information is, of course, also freely available in the deposited sequence data.

We have also removed the relevant section on AMR from the discussion, for the same reason.

## Discussion:

- The discussion would benefit from restructuring, e.g. according to the recommendations of Docherty and Smith: BMJ 1999;318:1224-5 (summary of findings; comparison with previous literature ; strengths and limitations ; meaning of the study and understanding possible mechanism; implications for practice, policy and future research).

**Authors’ response:** Thank you for this suggestion. We have focused and restructured the discussion section, as above

- Suggestion to shorten the summary of the evidence in the introduction and move the details on current evidence on NTS transmission to the discussion, so you can discuss all evidence at once here and compare your data with it.

**Authors’ response:** Thank you for this suggestion. We have shortened the introduction, and focused and restructured the discussion section, as above

- Line 412 - 415: "While we failed ... in this area". Do you think the technical challenges with isolation of S. Typhi might have affected the yield of NTS too? If so, do you think it might have caused a bias towards less iNTS isolation compared to non-invasive NTS? It would be interesting to know how many stool samples you finally obtained per household member, as you expect incremental yield if multiple stool samples are analysed.

**Authors’ response:** As above, we do not believe this is the case. The sampling distribution is now comprehensively documented in the additional material provided in the maps and new table. The information that our NTS isolation rate is in keeping with previous literature is already included in the manuscript. We have added a comment on the rarity of healthy stool carriage of S.Typhi compared to iNTS in our setting.

W.r.t samples per individual, as is stated in the methods section, there was one sample pot submitted per individual, containing pooled samples over 24 hours. Although we encouraged individuals to pool samples from up to 3 separate motions within that time period, we did not ascertain each individual’s stool frequency, or how many individual stool motions, and of what volume/proportion, were pooled from each individual, since we judged that this was not feasible to assess in a situation where people are using unlit crouching long-drop toilets.

- Line 420: Is it possible to assess the hypothesis on ST313 Lineage 3 based on your WGS data?

**Authors’ response:** Thankyou. This comment has been removed, in the course of removing the more general discussion of antimicrobial resistance, which was not felt to be directly pertinent to the hypothesis of the paper.

## Editorial

- Line 78: suggestion to harmonize typhoidal and non-typhoidal (both without capital)
- Line 97-99: "In Sub-Saharan Africa (SSA), however, the epidemiological picture... carries an estimated case-fatality of 14.7%." Split sentence to improve readability.
- Line 101-104: "Two Salmonella serotypes ...  ST11 being the most frequently identified." Split sentence to improve readability. Furthermore the list with countries from which data on the serotype distribution are available is incomplete. To avoid the suggestion that enteritidis and typhimurium are not the main serotypes in other, non-listed sub-Saharan African countries, either complete the list or use more general wording.
- Line 305: Suggestion to replace "associated with different households" by "sampled in different households"
- Line 329 - 334 "Striking examples ... in the same households". Hard to read, please split in multiple sentences and rephrase.
- Line 364: "52 genomes" sounds a bit strange to me
- Line 365: Please harmonize, either addition of s to abbreviation for plural or not ("9 ST313s, 3 ST3257")
- Line 397 - 399: "The lack of overlap ... very striking". Please correct sentence.
- Line 403-406: "The presence of the same STs... from iNTS cases". Please split sentences and rephrase.

**Authors’ response:** Thankyou. These have all been addressed and amended as appropriate

# Reviewer #2 (Kirsten E Wiens, IHME)

## Summary

This is an important paper that provides evidence for household transmission of invasive Salmonella infections in Blantyre, Malawi, and no evidence for household environmental or animal reservoirs. This is consistent with previous work in Kenya, Burkina Faso, and The Gambia, and could support a future study that investigates Salmonella household transmission prospectively.

**Authors’ response:**we thank reviewer 2 for noting the importance of these findings, and would additionally point out that the work is not only consistent with that from Kenya and Burkina Faso, but extends it considerably by adding environmental sampling which has not bee undertaken in those studies. We agree that these findings could now pave the way for a prospective longitudinal transmission study.

As described above, there are several areas where additional details are needed, including:

1) Further detail on sample collection and population in a new table and in the methods.

**Authors’ response**: Thank you. This was a recommendation in common with review 1, and has been done as above

2) Further detail on isolate diversity/similarity by household (Figure 2) and sampling sources (new table corresponding current Table 2).

**Authors’ response**: Thank you. This was a recommendation that overlapped with with review 1, and further details have been provided as above, including a panel-figure of 2 maps, and a new demographics table

3) Given the limitations of this study, discussion of what additional information/studies are needed to inform iNTS mitigation.

**Authors’ response**: Thank you. This has been more clearly outlined in the conclusions and recommendations, including the need for longitudinal studies to resolve transmission routes.

## Methods

The objective of this study—to examine potential household sources of invasive non-typhoidal Salmonella (iNTS) infection—is clearly laid out, and the approach of examining diversity of Salmonella isolates from the environment, animals, and humans living in households of index iNTS cases is appropriate to address it.

A strength of the approach is extensive environmental sampling, which supports their conclusions that household environmental sources harbor diverse NTS strains but are not major sources of iNTS. A limitation of the design (which the authors discuss) is that in was not feasible to examine community, food, and/or water sources. Another unknown is timing of infections given the sampling delay of up to 2 weeks following index case enrollment, and it’s possible that there were additional sources of infection/matched isolates that were missed.

**Authors’ response**: Thank you for this recognition of the strengths of our approach, and for noting that we have already identified and discussed the limitations of the design.

In addition, there are several areas where more detail is needed on the study population and sample collection:

- How was household membership defined?

People who usually sleep in the household.

- It would be helpful to include a table summarizing characteristics of the recruited participants, including age group, sex, household size, symptoms in index cases, timing between index enrollment and household sampling, and any other pertinent information from the socio-demographic questionnaire.

**Authors’ response**: Thank you. This was a recommendation that overlapped with with review 1, and further details have been provided as above in the new demographic table (new Table 1) and in the text. The limitation of delay to sampling is addressed more specifically in the text.

- The authors collect 3 consecutive stool samples from humans, 1 from animals, and use environmental “boot-sock” sampling. Recovery rate of Salmonella isolates is similar between human, animal, and environmental sources in aggregate, but I’m unsure if this alone indicates that sampling was sufficient. Are there other measures or control sequences that could be used to compare the level of sampling between households and sources?

**Authors’ response**: Thank you. The new table and maps now clearly demonstrate the breadth and even spread of sampling and of sample positivity across the whole study area. We have added commentary in the text, as per our response to reviewer one above, to demonstrate that

a) sampling was highly representative of community demographic structure
b) sampling was evenly spread across the whole study-area
c) sampling was comprehensive in coverage considering expected household size and age-structure

- Line 256. “Sample positivity was…similar across different household categories.” In Table 1, positivity rates vary from 0 to 28% between different categories—what do the authors mean in this sentence?

**Authors’ response**: Thank you. Like reviewer 1, the reviewer seems to be mistakenly referring to *household* positivity according to category.

The text reads “Overall, ***sample*** positivity was similar for human, animal and bootsock environment samples (human stool 16/491, 3.3%; animals 4/110, 3.6%; bootsocks 23/620, 3.8%), and across different household categories (Table 1)”. (our italics for clarity)

This statement clearly refers to the rate of *sample* positivity, which is 3.2%, 2.3%, 3.2% and 5.5% across the 4 household categories. This is an important point demonstrating the robustness of culture methods (see other points raised by reviewer 1). We have therefore made no change to the manuscript.

- Line 257. Animal sampling was from 59% of households. Did the rest of the households not have animals, or not perform animal sampling?

**Authors’ response**: Thank you for raising this question. Animal ownership is relatively low in urban Blantyre, as conditions are very crowded. The reason for non-sampling of animals in some households is that they did not own animals. This has been mentioned in the manuscript

## Results

Overall, the tables and figures are clear and match the analysis plan. Two areas for improvement:

- In Figure 1, it would be helpful to additionally visualize the household similarity among NTS not linked to invasive disease (results in paragraph 327-338). For example, delineating households where environmental, animal and/or healthy human samples match using purple, green, or dashed lines (as was done in black and red for the matched invasive disease isolates).

**Authors’ response**: Thank you for this helpful suggestion. This has been added to the new Figure 2.

- The individual-level isolate data provided in Table 2 is great, but it’s also hard to digest all this information at once. It would be helpful to have an additional table summarizing the serogroups by source of infection. For example: for each sequence type/serovar/serogroup (in rows) what number & percent were isolated from each household sample type (in columns).

**Authors’ response**: Thank you for this helpful suggestion. As there was a lot of overlap between old Table 2 and Supplmentary Table 1, we have replaced old Table 2 with the simpler table you suggested (now Table 3), and all the per-isolate information is now available in Supplementary Table 1.

## Discussion

The conclusions that this study provides evidence for household transmission of iNTS and no evidence for animal or environmental household reservoirs are supported.

The evidence for person-to-person transmission comes from only 2 of the 28 index iNTS cases, and not all potential sources including food, water, and community sources could be sampled (which the authors discuss in the limitations). So, the conclusion on lines 374-375 that “Our findings support the hypothesis that humans are the primary reservoir for iNTS in sub-Saharan Africa” is not strongly supported, and the authors may consider rephrasing.

**Authors’ response**: Thank you for this comment, which overlaps with comments from reviewer 1, as discussed above. We acknowledge that these cross-sectional data are not able to distinguish between sources and reservoirs, as that would require a longitudinal study. Without longitudinal data it is also not possible to definitively prove transmission routes.

However, we have added much more comprehensive and robust cross-sectional data to the field than has previously been available, which greatly strengthens the evidence for “lack of evidence” from animals or environment, so we feel that the language stating that our findings are ‘consistent with’ or ‘support’ a hypothesis lf human transmission remains entirely accurate and valid.

We have therefore modified the conclusion to say that the “**findings support a hypothesis of human to human transmission”.**

We have also added this wording to the title

In addition, the paper would be strengthened by:

- Including the limitation of potential delays between infection/exposure and sampling.

**Authors’ response**: Thank you for this, we have added a comment as suggested (also requested by reviewer 1)

- Discussing what is needed in future studies to test the hypothesis that healthy humans were the source of iNTS infections in the household (e.g., prospective sampling) and what the results of such a study would mean for iNTS prevention and control.

**Authors’ response**: Thank you for this suggestion, we have included this in the discussion, particularly the need for longitudinal studies to study dynamic transmission sources and reservoirs over time.

# Editorial comment:

The paper is mostly very well written. Some comments:

- Please make sure to define all acronyms the first time they are presented in the text.

- Suggest including study location (Blantyre, Malawi) and dates (Mar 2015-Oct 2016) in the abstract.

We have done so.

- Suggest replacing “Africa” in the title with “Malawi”.

**Authors’ response**: Thank you, we have done these. We have made small additional modifications to the abstract to keep within the required word count following requested amendments. Please note that we have made a change to the title, in line with the discussions above.

# Reviewer #3:

## Summary

Koolman et al present data on *Salmonella* isolates collected from people, household members, animals, and the household environmental of invasive *Salmonellosis* cases and their matched controls households in Blantyre, Malawi. They report on both invasive non-typhoidal *Salmonella* and typhoid cases and matched controls. They also report sequence type, serovars, and serogroup. Finally, they present results of whole genome sequencing of the *Salmonella* isolates collected. The study design, robust and novel sampling, and the clinical and public health importance of this issue make this a valuable contribution to the literature. However, some key study concepts need to be clarified in this manuscript in order to maximize the impact of this study. Addressing these and other concerns listed below would greatly improve this important work. Additional minor concerns also listed below.

## MAJOR EDITS/CONCERNS

My primary concern with the manuscript is how the study outcome has been described and written about. It appears the authors enrolled iNTS and typhoid cases, that is, patients with iNTS and typhoid disease (see lines 31, 107). However, iNTS is also referred to as a cause of infection rather than the result of infection (see lines 120, 374, 441-442). For example, I believe line 374 should say humans are the primary reservoir for iNTS *causing Salmonella*, not reservoir for iNTS. Please use terminology consistently throughout the manuscript, being explicit about what is the pathogen/etiology versus the disease/symptoms.

## **Authors’ response**: Thank you for this correct observation. We have amended this throughout the manuscript

The Introduction should be edited to clearly explain the relationship between these terms, as well as to introduce the concepts of sequence type, serovar, and serogroups of *S.*, and to describe the symptoms and clinical presentation of iNTS and typhoid versus non-invasive *S.* infection. Also, please be sure to update the study aims listed in lines 128-134 appropriately. See also lines 78-82, 260-261.

## **Authors’ response**: Thank you. This was a concern in common with other reviewers, and the introduction and abstract has been accordingly restructured for greater clarity

## MINOR EDITS/CONCERNS

Please define abbreviations at first use.
Line 27: isolates from symptomatic and healthy people

**Authors’ response**: Thank you, we have added “index cases”

Line 92-94: Add additional detail on incubation period, how long S. typhi survives in environment, etc.

**Authors’ response**: this is discussed in response to similar comments from reviewer1 and 2, and amendments made to the manuscript

Line 109: environmental factors could also lead to seasonal patterns that have nothing to do with animal reservoirs, e.g. more person-to-person contact during cold weather and therefore increased person-to-person transmission.

**Authors’ response**: Thank you, this is true. However, as discussed above, without longitudinal data the details of possible transmission dynamics are beyond the scope of this manuscript

Line 128: Authors list the study goal as identifying the reservoir of Salmonella strains responsible for invasive disease. The authors do not list this, but this study also answers the more fundamental question of what strains of Salmonella are people infected with when they have iNTS and typhoid symptoms/disease?

**Authors’ response**: Thank you. Although we do here identify the strains responsible for disease in 60 cases, there have been much much larger studies from our unit numbering thousands of cases, which have investigated this much more comprehensively and which are cited in the manuscript, so we do not feel that the data in this paper contributed in a major way to this aspect of current knowledge.

Supplemental figure would benefit from footnotes or other method to provide additional information about abbreviations.

**Authors’ response**: Thank you, we have reviewed and amended where needed

## Methods

Study would have benefited from enrolling additional households (cases and controls). But statistical comparisons were not made between groups so small sample size not a concern from a statistical perspective.

**Authors’ response**: Thank you, we have made no change in response to this comment, this is mainly a descriptive study
