# Supplementary material for: Case-control investigation of invasive Salmonella disease in Malawi reveals no evidence of environmental or animal transmission of invasive strains, and supports human to human transmission
Source: PLoS Negl Trop Dis. 2022 Dec 12;16(12):e0010982. doi: 10.1371/journal.pntd.0010982 (PMC9779717; doi:10.1371/journal.pntd.0010982)
Supplement: Attachment — Submitted filename: 2022.10.27 response to review.docx [file pntd.0010982.s005.docx]

Dear Dr Ashton,

Thank you very much for submitting your manuscript "Case-control investigation of invasive Salmonella disease in Malawi reveals no evidence of environmental or animal transmission of invasive strains, and supports human to human transmission." for consideration at PLOS Neglected Tropical Diseases. As with all papers reviewed by the journal, your manuscript was reviewed by members of the editorial board and by several independent reviewers. The reviewers appreciated the attention to an important topic. Based on the reviews, we are likely to accept this manuscript for publication, providing that you modify the manuscript according to the review recommendations.

Please prepare and submit your revised manuscript within 30 days. If you anticipate any delay, please let us know the expected resubmission date by replying to this email. 

When you are ready to resubmit, please upload the following:

[1] A letter containing a detailed list of your responses to all review comments, and a description of the changes you have made in the manuscript.
Please note while forming your response, if your article is accepted, you may have the opportunity to make the peer review history publicly available. The record will include editor decision letters (with reviews) and your responses to reviewer comments. If eligible, we will contact you to opt in or out

[2] Two versions of the revised manuscript: one with either highlights or tracked changes denoting where the text has been changed; the other a clean version (uploaded as the manuscript file).

Important additional instructions are given below your reviewer comments.

Thank you again for your submission to our journal. We hope that our editorial process has been constructive so far, and we welcome your feedback at any time. Please don't hesitate to contact us if you have any questions or comments.

Sincerely,

Andrew S. Azman
Section Editor
PLOS Neglected Tropical Diseases

***********************

Reviewer's Responses to Questions

<b>Key Review Criteria Required for Acceptance?</b></br></br>
As you describe the new analyses required for acceptance, please consider the following:</br></br>

<b>Methods</b></br></br>
-Are the objectives of the study clearly articulated with a clear testable hypothesis stated?</br>
-Is the study design appropriate to address the stated objectives?</br>
-Is the population clearly described and appropriate for the hypothesis being tested?</br>
-Is the sample size sufficient to ensure adequate power to address the hypothesis being tested?</br>
-Were correct statistical analysis used to support conclusions?</br>
-Are there concerns about ethical or regulatory requirements being met?</br></br>

Reviewer #1: No remaining pertinent comments on the methods in the revised version

Reviewer #2: - The definition for household membership was provided in response to my comment, but not added to the methods. Please add this definition to the methods.

Response: this has been added on L159-160.

- I now understand what the authors mean in the sentence “Sample positivity was…similar across different household categories” based on the authors' response, but the text in the manuscript is still unclear. I suggest editing this sentence to clarify what “household categories” refers to and why having comparable sample positivity is important.

Response: We have clarified this on L278-279.

Reviewer #3: With these revisions, this study and manuscript appear to satisfy these criteria. The overarching study goal/hypothesis is clear, and the study design and population appropriate. Explicit statistical hypothesis testing was not undertaken, so sample size is not a major concern. No ethical concerns.

--------------------

<b>Results</b></br></br>
-Does the analysis presented match the analysis plan?</br>
-Are the results clearly and completely presented?</br>
-Are the figures (Tables, Images) of sufficient quality for clarity?</br></br>

Reviewer #1: •  Table 1: Please add a legend explaining the abbreviations: NA is not applicable or not available?

Response: We have clarified this, as suggested
•       Figure 1: I suggest the authors to reconsider how the data can be better visualized to be more informative.
o       Part A: If feasible, I suggest harmonization with categories used in table 2: e.g. distinguish between index (typhoid fever), index (iNTS) and control households by using small pie charts with size adjusted to number of households per area. If not feasible, numbers stating the numbers of households might be more informative than the bubbles that are now used.
o       Part B: It is very hard for the reader to grasp the data here, since the information is scattered around the map instead of geographically visualized. The times 2 or 3 to indicate multiple isolates from the same sequence type is a bit lost amidst all the labels. Please reconsider how you can visualize this more clearly without losing the granularity on source and ST. Maybe working with multiple chloropleth maps (one per source and one for invasive + household member) might be a solution.

Response: we would like to thank the reviewer for engaging with our figure. We agree that it was rather “busy”, and to resolve this we have split the typhoid fever households and the iNTS households into separate maps, please see new Figure 1.

•       Line 321-322 (legend figure 2): “Isolates of the same ST from different niches within the same household are indicated by purple, green and magenta outlines.” It is not clear to me how I should interpret this. Can you please explain in more detail to facilitate a better understanding?

Response: We have expanded our explanation of the meaning of the “pie slice” outlines in the legend of Figure 2.

•       Line 338: I suggest the authors to specify that serotypes mentioned in the following paragraphs were determined in silico.

Response: we have clarified this on the new line 355.

Reviewer #2: (No Response)

Reviewer #3: This version of the manuscript is much clearer than the previous version. Necessary tables and figures are provided and labeled appropriately. The analysis appears to match the original plan.

--------------------

<b>Conclusions</b></br></br>
-Are the conclusions supported by the data presented?</br>
-Are the limitations of analysis clearly described?</br>
-Do the authors discuss how these data can be helpful to advance our understanding of the topic under study?</br>
-Is public health relevance addressed?</br></br>

Reviewer #1: •  Line 450 – 452: The high positivity rate on household level in typhoid fever index- and control households compared to iNTS index- and control households is an interesting finding and largely resulted from a difference in yield of animal and environmental samples. Do the authors have any hypothesis/potential explanation for this finding? And what about the absence of Salmonella in fecal samples of iNTS-control households?

Response: We also noted this difference, but we do not have any explanation that can be backed up by data. Therefore, we are not keen to speculate on potential causes for this association in the manuscript.

•       Limitations:
o       The authors now explicitly state that none of the household members was treated with antibiotics for iNTS disease, so that no household members had to be excluded for this reason. However, if I understood it correctly, it is possible that some household members were on antibiotics at the moment of sampling. If so, I recommend mentioning this as a limitation.

Response: we have added a mention of this limitation on L531-533 of the revised manuscript.

o       The authors explained in their rebuttal letter that it was not feasible to collect data on stool frequency/consistency of household members or to know how many stool motions were sampled per household member. Since this might impact the yield, I recommend adding this to the limitation section.

Response: we have added a mention of this limitation to L535-537 of the revised manuscript.

Reviewer #2: - Suggest rephrasing lines 75-77 to something like: “Our findings support a hypothesis that invasive Salmonella infections are transmitted within households from non-animal or environmental sources.”

Response: The reviewers suggested re-phrase ignores the positive finding of the ST313/ST3257 in the stool of household members. Additionally, the other two reviewers were happy with this phrasing so we propose to leave this section as it is.

Reviewer #3: The strengths, weaknesses, and limitations of available data are thoroughly discussed. Conclusions are well aligned with results and don't over-generalize. Findings are well situated in our existing knowledge of the topic and clearly describe what this study adds to our understanding. Authors have also done a good job of highlighting what understanding this study design is not able to support (ie cross sectional so not able to definitely show transmission pathways).

--------------------

<b>Editorial and Data Presentation Modifications?</b></br><br/>
Use  this section for editorial suggestions as well as relatively minor modifications of existing data that would enhance clarity. If the only modifications needed are minor and/or editorial, you may wish to recommend “Minor Revision” or “Accept”.

Reviewer #1: (No Response)

Reviewer #2: - Suggest removing “, and supports human to human transmission” from the title.

Response: The reviewers suggested re-phrase ignores the positive finding of the ST313/ST3257 in the stool of household members. Additionally, the other two reviewers were happy with this phrasing so we propose to leave this section as it is.

- The “tracked changes” version of the manuscript does not correspond to the clean version of the manuscript provided. Please upload a manuscript that shows what changes were made to arrive at the revised manuscript.

Response: Apologies for that. We’re not quite sure how to provide that along with this revision, but if the journal staff can assist, we can forward to email (via the journal staff).

Reviewer #3: (No Response)

--------------------

<b>Summary and General Comments</b></br></br>
Use  this section to provide overall comments, discuss strengths/weaknesses of the study, novelty, significance, general execution and scholarship. You may also include additional comments for the author, including concerns about dual publication, research ethics, or publication ethics. If requesting major revision, please articulate the new experiments that are needed.

Reviewer #1: o  The manuscript has much improved after restructuring and addition/modification of tables/figures. Congratulations to the authors for their thorough revision!
o       There are still some typos or syntax errors due to the textual modifications. I advise the authors to scan the manuscript one more time to correct these.

Reviewer #2: I thank the authors for their extensive revisions and detailed responses. My remaining comments are minor and refer to areas of the text where further clarity would be helpful.

Reviewer #3: Overall edits are very good. This updated version of the manuscript is much clearer and persuasive while being explicit about what conclusions are and are not supported by the findings. The Author Summary is excellent.

--------------------

PLOS authors have the option to publish the peer review history of their article (<a href="<https://journals.plos.org/plosntds/s/editorial-and-peer-review-process#loc-peer-review-history>" target="_blank">what does this mean?</a>). If published, this will include your full peer review and any attached files.<br><br>

If you choose “no”, your identity will remain anonymous but your review may still be made public.<br><br>

<b>Do you want your identity to be public for this peer review?</b> For information about this choice, including consent withdrawal, please see our <a href="<https://www.plos.org/privacy-policy>" target="_blank">Privacy Policy</a>.

Reviewer #1: No

Reviewer #2: Yes: Kirsten E. Wiens

Reviewer #3: No


Figure Files:
While revising your submission, please upload your figure files to the Preflight Analysis and Conversion Engine (PACE) digital diagnostic tool, [https://pacev2.apexcovantage.com](https://pacev2.apexcovantage.com/). PACE helps ensure that figures meet PLOS requirements. To use PACE, you must first register as a user. Then, login and navigate to the UPLOAD tab, where you will find detailed instructions on how to use the tool. If you encounter any issues or have any questions when using PACE, please email us at [figures@plos.org](mailto:figures@plos.org).

Data Requirements:
Please note that, as a condition of publication, PLOS' data policy requires that you make available all data used to draw the conclusions outlined in your manuscript. Data must be deposited in an appropriate repository, included within the body of the manuscript, or uploaded as supporting information. This includes all numerical values that were used to generate graphs, histograms etc.. For an example see here: <http://www.plosbiology.org/article/info%3Adoi%2F10.1371%2Fjournal.pbio.1001908#s5>.

Reproducibility:
To enhance the reproducibility of your results, we recommend that you deposit your laboratory protocols in [protocols.io](http://protocols.io/), where a protocol can be assigned its own identifier (DOI) such that it can be cited independently in the future. Additionally, PLOS ONE offers an option to publish peer-reviewed clinical study protocols. Read more information on sharing protocols at <https://plos.org/protocols?utm_medium=editorial-email&utm_source=authorletters&utm_campaign=protocols>

References
Please review your reference list to ensure that it is complete and correct. If you have cited papers that have been retracted, please include the rationale for doing so in the manuscript text, or remove these references and replace them with relevant current references. Any changes to the reference list should be mentioned in the rebuttal letter that accompanies your revised manuscript. If you need to cite a retracted article, indicate the article's retracted status in the References list and also include a citation and full reference for the retraction notice.
